# Reinterpretation in visual imagery is possible without visual cues: a validation of previous research

**DOI:** 10.1007/s00426-017-0956-5

**Published:** 2017-12-14

**Authors:** Kevin L. Kamermans, Wim Pouw, Fred W. Mast, Fred Paas

**Affiliations:** 1 Department of Psychology, Education and Child Studies, Erasmus University Rotterdam, Rotterdam, The Netherlands; 2 Department of Psychological Sciences, University of Connecticut, Storrs, USA; 3 Department of Psychology, University of Bern, Bern, Switzerland; 4 Early Start Research Institute, University of Wollongong, Wollongong, Australia

**Keywords:** Visual imagery, Visual bistability, Haptic perception, Mental rotation, Imagery Debate

## Abstract

Is visual reinterpretation of bistable figures (e.g., duck/rabbit figure) in visual imagery possible? Current consensus suggests that it is in principle possible because of converging evidence of quasi-pictorial functioning of visual imagery. Yet, studies that have directly tested and found evidence for reinterpretation in visual imagery, allow for the possibility that reinterpretation was already achieved during memorization of the figure(s). One study resolved this issue, providing evidence for reinterpretation in visual imagery (Mast and Kosslyn, Cognition 86:57–70, 2002). However, participants in that study performed reinterpretations with aid of visual cues. Hence, reinterpretation was not performed with mental imagery alone. Therefore, in this study we assessed the possibility of reinterpretation without visual support. We further explored the possible role of haptic cues to assess the multimodal nature of mental imagery. Fifty-three participants were consecutively presented three to be remembered bistable 2-D figures (reinterpretable when rotated 180°), two of which were visually inspected and one was explored hapticly. After memorization of the figures, a visually bistable exemplar figure was presented to ensure understanding of the concept of visual bistability. During recall, 11 participants (out of 36; 30.6%) who did not spot bistability during memorization successfully performed reinterpretations when instructed to mentally rotate their visual image, but additional haptic cues during mental imagery did not inflate reinterpretation ability. This study validates previous findings that reinterpretation in visual imagery is possible.

## Introduction

Although visual imagery is phenomenally familiar to most individuals, its psychological nature remains elusive. One of the central questions in what was dubbed “The Imagery Debate” concerns the structural resemblance between visual imagery and visual perception (Block 1981; Kosslyn 1994; Pylyshyn 2002; Tye 2000). On the one hand, it was argued that visual imagery operates on amodal propositional encodings that are transduced from perception and, therefore, functionally independent from (ongoing) constraints of visual perception (Pylyshyn 2002). On the other hand, it was shown that typical constraints of visual perception remain present in imagery (e.g., Shepard and Metzler 1971), which fueled the idea that visual imagery shares common mechanisms with visual perception. Although, there is still much discussion about the degree of resemblance between perception and imagery (e.g., Foglia and O’Regan 2015; Pearson and Kosslyn 2015), and the role of top-down amodal processes (e.g., Langland-Hassan 2015), there is a general consensus that imagery does not (only or necessarily) operate on amodal propositional encodings as was proposed by one of the main contenders of the classic imagery debate (Pylyshyn 2002). This consensus has been reached, in part, through behavioral evidence that suggests that visual imagery functions like visual perception (Kosslyn 1973; Shepard and Metzler 1971) in conjunction with neuroscientific evidence for iconic resemblance in neural organizations associated with visual perception (i.e., retinotopic representations; for an overview see Pearson and Kosslyn 2015).

Research that fueled the consensus that visual imagery does not only function on symbolic re-descriptions of visual information, is concerned with the possibility of reinterpretation of visually bistable figures (e.g., duck/rabbit figure by Jastrow 1899, p. 312) in visual imagery. Recent studies have argued that this is indeed possible (e.g., Peterson 1993; Mast and Kosslyn 2002), in contrast to a descriptivist account of visual imagery which argued for its impossibility (Pylyshyn 2002). However, due to some methodological loose ends there is a need for more empirical research to further buttress reinterpretability in visual imagery. This study aims to provide a validation of previous findings that is currently missing, but necessary as to reach such empirical consensus. Furthermore, we aim to assess whether cross-modal information via haptic perception can support visual imagery processes.

### Reinterpretation in visual imagery

Bistable figures have two interpretations (e.g., duck/rabbit figure; Jastrow 1900; Jensen and Mathewson 2011; Mitroff, Sobel, & Gopnik, 2006). Reinterpreting a bistable figure in *visual perception* involves attaining a new percept (e.g., rabbit) that visually dominates over the initial percept (e.g., duck) of an object. Attaining a new percept can be achieved through visual reinspection of the figure that fosters detection of relevant spatial correspondences between figure and an alternate novel interpretation. The common approach for testing reinterpretation *in visual imagery* is to assess whether an object Z (e.g., duck/rabbit figure) that is visually perceived as an X (e.g., rabbit) can be reinterpreted when recalled from memory in visual imagery as being a Y (e.g., duck). In other words, spatial correspondences between the imagined figure and its novel interpretation are detected in visual imagery.

Early phenomenological characterizations of visual imagery held that visual reinterpretation cannot be a general feature of imagery, since images are typically created by the imaginer (Sartre 1940; see also Dalla Barba, Rosenthal, & Visetti, 2002). Thus, discovery of a novel interpretation is unlikely since a self-invoked visual image is bound to the interpretation it was given when generating the image intentionally (Chambers and Reisberg 1985). If this is correct, visual imagery functions in a sense like descriptions, in that the content of visual images is tied to a *mode of presentation* transferred from one’s intentions (Langland-Hassan 2015). As such, the functioning of visual images goes beyond the sensory image itself; they have a frame of reference (Chambers and Reisberg 1985), and are “images under a description” (Fodor 1975, p. 190l). Thus, on this descriptive view of visual images, it is predicted that discovery of novel interpretations of images in visual imagery (i.e., reinterpretation) is not possible (Chambers and Reisberg 1985; Pylyshyn 2002; Fodor 1975).

If reinterpretation is possible, it would support a core idea of the *Quasi-pictorial Account* of visual imagery (Kosslyn, Thompson, & Ganis, 2006). One of its core ideas is that visual imagery functions like visually perceiving pictures (e.g., drawings, diagrams). Pictures do not have a fixed meaning—their meaning is dependent (amongst others) on an interpreter detecting relevant correspondences with other objects (Kulvicki 2014). Analogously, visual images may be like pictures, such that visual images preserve and bring forth the spatial properties of a memorized object (Z), without fixing the meaning that was initially assigned to the object (e.g., Z as X). Therefore, the quasi-pictorial account predicts that visual imagery would allow for detection of novel meanings in mental images.

It has to be noted that the possibility of reinterpretation in visual imagery does not necessarily support all core ideas of the quasi-pictorial account (e.g., Thompson 2007), nor do we think it is necessarily the only account that is in par with it (e.g., Langland-Hassan 2015; Thomas 1999).^1^ Rather, the possibility of reinterpretation would indicate that visual imagery does not necessarily function as descriptions, and allow for perceptual acts similar to pictorial representations.

### Empirical evidence for reinterpretation in visual imagery

Is reinterpretation in visual imagery possible? The first landmark study by Chambers and Reisberg (1985) suggested a negative answer. In their study, participants were *first* familiarized with bistable figures with several examples. Subsequently, participants were shown a novel figure; the classic duck/rabbit figure (Jastrow 1899). This duck/rabbit figure was presented briefly (i.e., 5 s) as to ensure that participants perceived only one interpretation instead of both. Results showed that none of the participants could reinterpret the figure in their visual imagery when told that it was bistable. In contrast, all participants detected the novel interpretation when drawing out their mental image on paper, suggesting that the failure to detect bistability in visual imagery was not due to poor memorization of the figure.

Finke, Pinker, and Farah (1989) expanded on previous findings by demonstrating that novel interpretations can be made in visual imagery by combining visually simple and highly familiar objects. Subjects were asked to imagine the capital letter D turned 90 degrees to the left and resting on top of the capital letter J, upon which they were able to see an umbrella in this new construal. Subsequent studies including more complex figures like the duck/rabbit figure showed that such figures are in fact reinterpretable in imagery (i.e., Brandimonte and Gerbino 1993; Chambers and Reisberg 1991; Hyman and Neisser 1991; Peterson, Kihlstrom, Rose, & Glisky, 1992). One of these studies using the duck/rabbit figure found that 40% of participants were capable of detecting the alternate interpretation in visual imagery (Peterson et al. 1992).

To make sense of the inconclusive findings on reinterpretation in visual imagery, Peterson and colleagues (1992) argued that the outcome of these studies depends on how congruent the bistable figure example is with respect to the test figure(s). That is, the example figure that is being used to familiarize participants with visual bistability needs to be reversed in a manner that is similar to the test figure(s) for reinterpretation to occur. This would explain the null-findings of Chambers and Reisberg (1985) who used bistability examples that required different reorientations than the duck/rabbit test figure to detect reversal (e.g., down-up reversal vs. front-to-back reversal). Other studies resolved this problem using more congruent bistability examples that required the same reversal strategies as the test figure, indeed leading to improved reinterpretation (Brandimonte and Gerbino 1993; Hyman and Neisser 1991; Peterson et al. 1992). With regards to the positive findings of Finke and colleagues, test stimuli involved very simple and highly memorized stimuli (i.e., alphabetic letters; symbolic representation) that do not directly compare to having to interpret and reinterpret iconic representations through visual imagery.

However, as identified by Mast and Kosslyn (2002), there is a methodological issue in all previous studies that preclude inferences about the possibility of reinterpretation in visual imagery. In the previous studies, example bistable figures are consistently demonstrated *before* participants have to memorize the test figures (e.g., Peterson et al. 1992). This could have possibly alerted participants about the presence of bistability in the upcoming test figures that they had to memorize. Indeed, Slezak (1991) has found that participants who were familiar with figure bistability before memorization of a second figure were able to detect bistability, but they were not able to do so for the first presented figure which was not preceded by a cue about bistability. In sum, providing a bistability example *before* test figures are presented renders it problematic to conclude whether participants attained the novel interpretation in their visual imagery or inadvertently already noticed (or were predisposed to be sensitive to) the bistability while perceiving the figure.

**Fig. 1:**
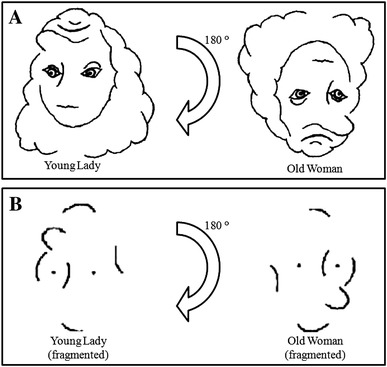
Bistable figure (**a**) and corresponding fragmented visual cues (**b**) as used in Mast and Kosslyn (2002). The young lady/old woman (**a**) was also used as an example bistable figure in the current study

Mast and Kosslyn (2002) evaded the previous methodological issue by not familiarizing participants with bistability. Instead, participants had to memorize the picture of the old woman/young lady depicted in Fig. 1. Bistability is hard to detect because the picture has to be rotated upside down to discover the second interpretation.

This was done by instructing participants to repeatedly draw the picture until they could draw it correctly from memory. Once memorized, participants were instructed to rotate their mental image upside down *with the aid of fragmented visual cues* from the original stimulus (also see Fig. 1). These visual cues were added as support during mental rotation because of the relatively complex nature of the old woman/young lady compared to previously used bistable figures (e.g., duck/rabbit). A total of 16 participants (out of 36 participants who did not discover bistability during memorization) were able to detect bistability in visual imagery combined with visual cues (i.e., fragmented version of Fig. 1). Importantly, additional participants were assigned to a separate control condition in which only the fragmented picture was presented. These participants did not perform above chance level when asked to choose from a list of possible interpretations. This finding excluded the possibility that being provided with the fragmented picture was sufficient for discovering the correct interpretation, suggesting that visual imagery played some functional role in reinterpretation with visual cues.

However, even though visual cues may not have been a *sufficient* condition for detecting a novel interpretation, it is possible that providing raw visual information directly is a *necessary* condition for making reinterpretation via visual imagery possible. Since it cannot be excluded that visual cues were a necessary condition for visual reinterpretation in Mast and Kosslyn's (2002) study, it is possible that the descriptivist claim that visual imagery alone does not allow for inspection of the raw sensory image still holds true (Chambers and Reisberg 1985; Fodor 1975; Pylyshyn 2002). This presumption is further reinforced by the finding that all participants in the control condition of Mast and Kosslyn's (2002) study reported that the fragmented pictures might represent a human face. As such, the fragmented cues were detailed enough to assign a semantic frame for further interpretation (i.e., face), thereby limiting the functional role that visual imagery alone may have played in successful reinterpretation. Therefore, based on the available evidence in the bistability detection paradigm, it is still an open question whether reinterpretation can be achieved through imagery processes alone, without assistance by visual cues.

In sum, two main issues should be resolved to validate previous studies that have found reinterpretation in visual imagery to be possible. First, it must be ensured that participants perceive only one interpretation by demonstrating bistability *after* memorization of the test figures has taken place. In studies with the most convincing evidence for bistability detection in visual imagery (excluding Mast and Kosslyn 2002), a bistability example was provided *before* presenting the test figures (e.g., Brandimonte and Gerbino 1993; Hyman and Neisser 1991; Peterson et al. 1992). Second, bistability detection should be tested in a paradigm that solicits a purely visual imagery process (i.e., without the aid of visual cues). Next, a further conceptual extension of previous research on reinterpretation is introduced.

### Multimodal imagery: visual imagery and haptic cues

We have argued that previous research allows for the possibility that visual imagery might only be possible, or is at least improved, because some direct visual information of the bistable figure is available. In extension of this possibility, the present study investigates whether such direct sensory cues (cf. visual cues in Mast and Kosslyn 2002) can be delivered through a non-visual modality as well; via haptic inspection (i.e., manual touch) of the bistable figure during visual imagery. Assessing whether imagery makes use of different sensory-systems dovetails with what Pearson and Kosslyn (2015, p. 10,091) have suggested to be one of the most pertinent questions today that has arisen out the aftermath of The Imagery Debate. Namely, “How many formats can the brain use? For example, do we have separate formats for motor, auditory, kinesthetic, and tactile information?”. If haptic cues indeed readily inform visual imagery, it would signal that mental imagery exploits multimodal information (i.e., visual and haptic).

That the visual perception system and the haptic perception system provide commensurable information has been found in a study by Held et al. (2011) who aimed to address Molyneux’s problem. Philosopher William Molyneux (1656–1698) posited a famous thought experiment: whether someone who was born blind and regained sight later in life would be able to visually recognize objects that were touched, but never seen before (Morgan 1977). Held et al. (2011) have negatively answered this question by showing that newly sighted people failed to match objects (sphere and cube) that they saw for the first time with what they had previously only felt. Yet, continued testing showed that people developed a multisensory awareness within a few days, successfully linking what they had previously only felt with what they were seeing. Thus, even though such sensori-motor knowledge is not present at birth, humans are naturally predisposed to *actively discover* meaningful invariances between information across different senses. Such transfer of information across the visual and haptic (i.e., touch) modalities has also been demonstrated in other studies (e.g., Wallraven, Bülthoff, Waterkamp, van Dam, & Gaißert, 2014; for a review see Lederman and Klatzky 2009). For example, when people are trained via touch to distinguish what category stimulus-objects belong to, they do not only show improvement when tested on haptic categorization, but also when tested on visual categorization (despite not having had any visual training). This transfer of information works vice versa, meaning that visual training also leads to improved haptic performance (Wallraven et al. 2014).

Considering the findings on multimodality in visual and haptic *perception*, it is not implausible that a similar visual-haptic multimodality exists for mental *imagery*. Indeed, studies on mental representations of hapticly perceived objects show considerable similarities with visual imagery effects. For example, mental scanning times have been shown to increase with spatial distance in both visual imagery and haptic imagery (Kosslyn 1973; Röder and Rösler 1998). The same holds true for mental rotation tasks (Dellantonio and Spagnolo 1990; Prather and Sathian 2002; Shepard and Metzler 1971). People take longer, both with visual and haptic stimuli, to judge the similarity between two objects depending on the angular disparity of those objects. Additionally, overlapping brain activation in visual areas is found during mental imagery for both visually and hapticly obtained information (De Volder et al. 2001). Given the ease of transfer of information between the haptic and visual system as shown by the studies above, we would expect that direct haptic cues of bistable figures during visual imagery will increase successful reinterpretation (as compared to no haptic cues). That concurrent haptic cues would interact with visual imagery processing ability is further supported by research indicating that haptic perception of pictorial 2-D figures is readily achieved, but only when subjects are aware of invariants that exist between haptic and visual-pictorial stimuli (Lederman, Klatzky, Chataway, & Summers, 1990). This is illustrated by congenitally blind subjects who do not have any visual experiences with pictorial representations and have much greater difficulties to interpret pictorial representations from haptic cues.

### Present study

The current study assessed whether reinterpretation is possible without visual cues (cf. Mast and Kosslyn 2002) and whether reinterpretation performance is improved by providing haptic cues. Notably, we used an example bistable figure that has a reversal strategy that is similar to the test figures (Peterson et al. 1992; cf.; Chambers and Reisberg 1985), and the participants were cued with this example of a bistable figure *after* memorization of the test figures (cf. Brandimonte and Gerbino 1993; Hyman and Neisser 1991; Peterson et al. 1992). Participants memorized the figures for 30 s.^2^ To assess the effect of haptic cues in detecting bistability in visual imagery, participants could freely touch the contours of, and rotate 2-D test figures by hand during visual imagery (Visual-Haptic condition), after having memorized the figure visually (without touch). Furthermore, we also included a Haptic Control condition, wherein participants hapticly explored one orientation of the test figure during memorization and the alternate upside down orientation during reinterpretation. This condition was included to control for the possibility that haptic cues were sufficient for establishing reinterpretation of the figures (cf. comparable to the control condition used by Mast and Kosslyn 2002). If visually imagining figures with concurrent haptic cues inflates successful reinterpretation relative to visual imagery without cues (Visual Only condition) and with haptic cues only (Haptic Control condition), this would suggest that the haptic system is able to work in concert with visual imagery. This would be an important finding as it would suggest that mental imagery does not operate on strictly “separate formats” for *visual and tactile* information (Pearson and Kosslyn 2015).

## Method

### Participants and design

Fifty-three participants were tested (36 female, *M*_age_ = 21.33 years, SD_age_ = 2.32 years, range 18–29 years). All participants were students from the Erasmus University Rotterdam who participated as part of a requirement of the Psychology program or voluntarily. Recruitment targeted native Dutch-speaking students (*N* = 31) and non-native international students (*N* = 22). For non-native students a translated English version of the Dutch instructions was used. All non-native participants were proficient in English, as they were enrolled in an international bachelor program instructed in English. In addition, no problems of instruction were observed with these participants during the experiment. This experiment was designed and conducted in accordance with the guidelines of the ethical committee of the Department of Psychology, Education, and Child Studies, at the Erasmus University Rotterdam.This study had a one-way within-subject design with condition as three-level factor (Visual Only vs. Visual-Haptic vs. Haptic Control) and bistability detection (no detection vs. detection) as main dependent variable. Each condition was assigned one unique bistable figure, i.e., one bistability detection trial per condition. Condition order, and figure-condition assignment was counterbalanced.

### Materials

#### Test figures and bistability example

**Fig. 2:**
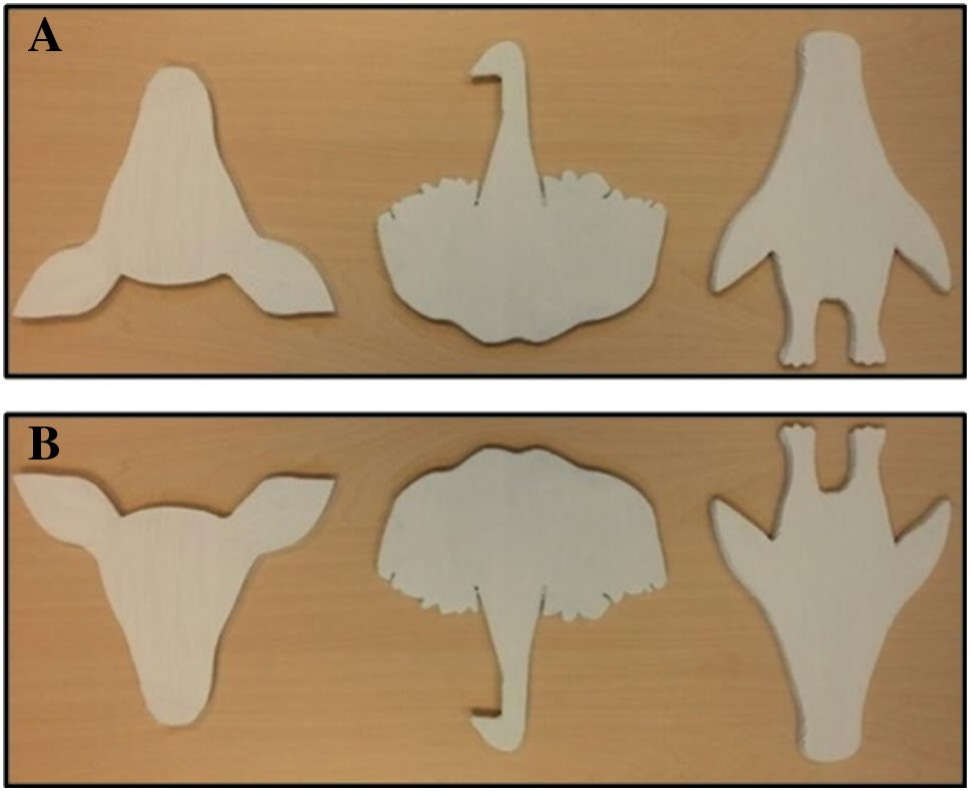
Test figures in body orientation (**a**) and head orientation (**b**). from left to right: seal/doe, swan/elephant, and penguin/giraffe

Three different bistable test figures depicted in Fig. 2 were designed based on the “Upside Down” campaign from Leo Burnett (2015) retrieved from Google images.

These figures were the seal/doe, swan/elephant, and penguin/giraffe. We selected these bistable test figures as they were simple enough to memorize, but at the same time also more complex to transform than other bistable figures (e.g., duck/rabbit) potentially reducing the amount of premature bistability detection during memorization. We also wanted to use novel figures that were not used in previous research as to make sure that participants (i.e., psychology students) were not already familiar with the figures. A simplified version of the old woman/young lady (Howard, 1982) depicted in Fig. 1 was printed on an A4 size sheet of paper and used for the bistability example phase (the test figures were presented as 2-D cutouts in Fig. 2; thickness = 0.5 cm, length = ca. 16 cm, width = ca. 21 cm). Each of the figures (test figures and example figure) had two readily perceivable interpretations. An alternate interpretation could be seen by rotating the Fig. 180° (i.e., upside down). In addition, the test figures of the animals shared a structural property, in that one interpretation always showed the *body* of an animal while the upside down interpretation showed the *head* of a different animal. The test figures were cut out of high-density foam sheets and had clear sensible edges so that participants could derive haptic sensory information from them.

#### Demographics and control questions

Upon completion of the experiment, participants filled out a short questionnaire. Participants were asked for their age, sex, and native language. Furthermore, to assess participants’ beliefs about the nature of the experiment they were asked about the perceived purpose of the experiment “What do you think was the purpose of the current study? (If you have no idea, no answer is necessary)”, and expectations “What do you think the researchers are expecting to discover with the current study? (If you have no idea, no answer is necessary)”.

#### Recording equipment

Answers given by participants were documented by the experimenter on a laptop computer. Performance was recorded using a JVC Everio GZ-MG130 camcorder, to ensure that data could be re-checked if necessary.

### Procedure

**Fig. 3:**
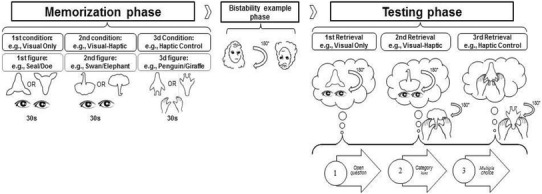
Flowchart depicting procedure of the experiment. Note, that order of condition and figure assigned to condition were counterbalanced. Orientation in which the figure was presented during the memorization phase was randomized, as well as the first presented orientation of the bistability example figure. In the testing phase, eye symbols within clouds means visual imagery, and hand symbols mean (concurrent) haptic perception

Participants were tested individually and were told at onset that they took part in a study about visual memory. The experiment consisted of three sequential phases that were conducted during a single test session. The three phases consisted of a memorization phase, bistability example phase, and a testing phase (see also Fig. 3).

#### Memorization phase

In the memorization phase three figures were presented successively. In this memorization phase, participants were explained that they will be presented with three different figures and that they had to form an accurate mental image of each of these figures because they would be tested on the material later on. Each figure was inspected for 30 s. For the Visual Only and Visual-Haptic condition, figures were presented visually and for the Haptic Control condition the figure was presented via manual touch only. In the Haptic Control condition, participants could bimanually inspect the cutout figure through two opening slots in a closed cardboard box. After each 30 s presentation of a figure, participants were asked what he/she had seen or felt (depending on condition). The experimenter noted down what participants detected in the figures. If participants reported perceiving two or more distinct interpretations of a figure during the memorization phase, the associated testing condition would be skipped. It could also occur that participants would only perceive the upside down interpretation of the figure (e.g., the figure was presented with the *head* orientation, but the participant reported an upside down interpretation corresponding to the *body* orientation). This occurred in 23 of 159 instances, and we exclude these from our analyses as to base our results on the most homogenous sample.^3^

#### Bistability example phase

After the memorization phase, participants were presented with the bistability example figure of the old woman/young lady (Fig. 1), with orientation of presentation randomized. It was explained that this drawing was an example of a bistable figure and contained two interpretations. Upon inspection, they were asked what they saw in the drawing. After participants reported what they saw, they were asked whether they could find the alternate interpretation in the drawing as well. If participants reported that they could not discover the bistability, they were instructed to turn the drawing upside down and encouraged to look for the alternate interpretation again. If they were still unable to perceive the bistability, the experimenter would point out the features of the alternate interpretation until they reported that they could see it. When participants reported noticing the bistability, they were asked to point out the features of both interpretations so that the experimenter could verify whether they had actually perceived the bistability. It is important to note that this bistability example of the old woman/young lady is structurally related to the testing figures in that they both have two distinct interpretations that are orientation specific and require the same upside down rotation reversal strategy. To reiterate, this example procedure was employed to make sure that participants were aware what visual bistability means, so that they effectively seek a novel interpretation in the test phase. Moreover, being familiar with visual bistability also fosters visual reinterpretation in direct visual perception of novel test figures (e.g., Mitroff et al. 2006).

#### Testing phase

Last, the testing phase was administered with the three different conditions. Participants were instructed to mentally retrieve an image of one particular figure from the memorization phase (order of retrieval counterbalanced). Depending on condition, participants retrieved the mental image either through visual imagery alone (Visual Only condition), or through visual imagery with haptic feedback by providing the relevant cutout of the figure during visual imagery using the cardboard box (Visual-Haptic condition), or through haptic reinspection alone using the cardboard box (Haptic Control condition). Once participants indicated that they generated (or hapticly inspected) the mental image, the experimenter would inform participants that this figure was bistable. Participants in the Visual Only- and Visual-Haptic condition were then instructed to “rotate their mental image 180 degrees upside down, just like the old woman/young lady, in order to detect the alternate interpretation”. Participants in the Visual-Haptic condition were also told to physically rotate the figure. In the Haptic Control condition, participants were only told to physically rotate the figure to detect bistability through haptic reinspection.

Participants were then asked three consecutive questions in each of the three testing conditions. With each question, more information was revealed by the experimenter regarding the correct interpretation (similar to Mast and Kosslyn 2002). First, participants were asked the *open question* if they could detect the alternate interpretation in their rotated mental image. If participants reported that they could not detect the alternate interpretation, the experimenter would continue with the next question. Second, participants were given the *category hint* that the alternate interpretation was an animal and were asked whether they could discover an animal in their rotated mental image. The category hint would be skipped by the experimenter if participants already reported an (incorrect) animal after the open question. Last, participants were given a *multiple choice* selection of four possible animal alternatives (see Table 3 in “Appendix” for the selected choices per orientation for each figure). The multiple choice question was always asked regardless of whether participants already reported a correct interpretation or not. This provided participants the opportunity to reconsider their answer when given a selection of possible alternative interpretations.

For each question, participants were informed that they would get as much time as they needed to provide an answer. Although not central to our research question, the experimenter also recorded the time until the participant reported an answer (Open 1st question, *M* = 42.21 s, SD = 29.43 s, range = 6–172 s; 2nd Question, *M* = 10.08 s, SD = 19.80 s, range = 0–123 s; 3rd question, *M* = 10.45 s, SD = 14.83 s, range = 0–77 s), or that he/she was unable to discover the alternate interpretation (Open 1st question, *M* = 83.11 s, SD = 50.40 s, range = 0–238 s; 2nd Question, *M* = 35.72 s, SD = 33.05 s, range = 0–103 s). During all three questions (*open question, category hint*, and *multiple choice*) in all three conditions, participants were instructed to keep their eyes closed to prevent gaze-induced disruption or modulation in visual imagery (e.g., Buchanan et al. 2015; Markson and Paterson 2009).

Concluding the experiment, participants were thanked for their participation and asked to fill out a short questionnaire containing the control questions. In cases where participants detected a correct alternate interpretation in one or more of the testing conditions, the experimenter would ask explicitly if he/she had noticed the alternate interpretation during memorization or newly discovered it in visual imagery.

### Performance and scoring

Performance was measured as a dichotomous-dependent variable (correct vs. incorrect). An interpretation given by a participant was considered correct in case this interpretation was also reported by another participant when visually perceiving the figure in that orientation in the memorization phase. For example, if a participant in our sample reported having perceived a cow for the *head* orientation of the seal/doe figure during the memorization phase, then this would be considered a valid interpretation for all participants when reported for that figure and orientation in the testing phase. As such, participants served (primarily) as their own raters in the current study, in contrast to more arbitrary post-hoc experimenter judgments used in previous research (e.g., Peterson et al. 1992). However, four other interpretations were regarded as correct after a post-hoc agreement between the first and second author that these were undeniably plausible visual interpretations for the given figure and its orientation (view Table 4 in “Appendix” for an overview of all correct [post-hoc] interpretations). Note that these post-hoc changes do not alter the interpretation of the results.

## Results

### Sample and data elimination per condition

A total of 42 out of 159 (3 × 53) instances of reinterpretation per image (i.e., trials) were eliminated from these descriptive statistics (10/53 haptic control; 17/53 visual only; 15/53 visual-haptic). Of these trials 19 out of 42 were removed because participants detected the bistability of the figure prematurely (3/19 haptic control; 7/19 visual only; 9/19 visual-haptic) during the memorization phase (i.e., reported two interpretations; one interpretation in the orientation presented, and another upside down interpretation corresponding to the 180 degree rotated orientation). The other 23 trials (7/23 haptic control; 10/23 visual only; 6/23 visual-haptic) were removed because they reported an interpretation during the memorization phase that corresponded to the upside down orientation from the participant’s perspective.

### Descriptives successful reinterpretation

Table 1 reports the descriptive statistics showing the occurrence and rate of successful reinterpretations per condition. The key dependent variable of interest here is the percentage of successful reinterpretations (i.e., participants who provided a correct alternative interpretation) in the visual imagery condition for the *open question* (i.e., asking to find the alternate interpretation without any hints).

**Table 1:** Performance for Each Question Type per Test Condition

Test condition	Open question	Category hint	Multiple choice
Visual only	11/36 (30.6%)	13/36 (36.1%)	22/36 (61.1%)
Visual-haptic	18/38 (47.4%)	16/38 (42.1%)	27/38 (71.1%)
Haptic control	14/43 (32.6%)	14/43 (32.6%)	30/43 (69.8%)
Total average scores	36.9%	36.9%	67.3%

### Differences in performance between testing conditions

Three confirmatory statistical tests were performed using a Bonferroni adjusted alpha level of 0.0167 per test (0.05/3). We hypothesized that haptic cues in the visual-haptic condition would lead to increased performance compared to the visual only condition and the haptic control condition. We compared the three test conditions (within-subjects) for the *open question*, the *category hint*, and the *multiple choice* question, with the key dependent variables of interest being the amount of successful reinterpretation in each test condition for the *open question*. For the *open question*, performance was 30.6% reinterpretation (*N* = 25) in the visual only condition, 47.4% reinterpretation (*N* = 25) in the Visual-haptic condition, and 32.6% reinterpretation (*N* = 25) in the haptic control condition. A Cochran’s *Q* test was performed, which tests differences in proportion for paired categorical data with more than two groups. A Cochran’s *Q* test showed that there was no statistically significant difference in successful reinterpretation between the different testing conditions, χ^2^(2) = 1.125, *p* = .570. Similarly, there were also no significant differences in performance found between testing conditions for the *category hint,*χ^2^(2) = 1.412, *p* = .494, nor for the *multiple choice* selection, χ^2^(2) = 2.471, *p* = .291. Thus, regardless of test condition, participants showed no improvement in performance for the *open question, category hint*, and *multiple choice* selection.^4^

### Differences in performance between figures

Using an alpha level of 0.05, we also looked at differences in performance for the *open question* between the three figures, regardless of test condition. Performance for the *open question* was 20% reinterpretation for the seal/doe figure, 68.6% reinterpretation for the swan/elephant figure, and 28.6% reinterpretation for the penguin/giraffe figure, regardless of test condition. A Cochran’s Q test revealed that there was a statistically significant difference in performance between the three testing figures, χ^2^(2) = 15.125, *p* < .001. Pairwise comparisons using a Bonferroni correction of *p* = .0167 (0.05/3) revealed no significant difference in performance for the seal/doe figure compared to the penguin/giraffe figure, χ^2^(1) = 0.692, *p* = .405. However, a pairwise comparison between the seal/doe figure and swan/elephant figure revealed a significant difference in performance, χ^2^(1) = 8.067, *p* = .005. In addition to this, a significant difference in performance was also found between the penguin/giraffe figure and swan/elephant figure, χ^2^(1) = 12.250, *p* < .001. Table 2 shows the performance for each figure per question type and test condition. We refer to the “Appendix” (Table 6) for a comprehensive table showing performance for both orientations of each figure (*head* and *body* orientation) for each subsequent question in all three testing conditions.

**Table 2:** Performance for each figure per question type and test condition

Question type test condition	Seal/Doe figure	Swan/elephant figure	Penguin/giraffe figure
**Open question**			
Visual only	2/11 (18.2%)	6/9 (66.7%)	3/16 (18.8%)
Visual-haptic	5/15 (33.3%)	8/11 (72.7%)	5/12 (41.7%)
Haptic control	2/14 (14.3%)	8/15 (53.3%)	4/14 (28.6%)
**Category hint**			
Visual only	2/11 (18.2%)	4/9 (44.4%)	7/16 (43.8%)
Visual-haptic	5/15 (33.3%)	5/11 (45.5%)	6/12 (50.0%)
Haptic control	2/14 (14.3%)	8/15 (53.3%)	4/14 (28.6%)
**Multiple choice**			
Visual imagery only	5/11 (45.5%)	5/9 (55.6%)	11/16 (68.8%)
Visual-haptic	8/15 (53.3%)	10/11 (90.9%)	9/12 (75.0%)
Haptic control	7/14 (50.0%)	13/15 (86.7%)	10/14 (71.4%)

The percentage of detection rates are shown for each separate figure regardless of presented orientation in the memorization/retrieval phase

## Discussion

The current study has two main findings. First, when controlling for premature bistability detection during memorization reinterpretation in visual imagery is possible without visual cues. Eleven out of the 36 participants (30.6%), who were not aware of bistability during memorization, reported a novel interpretation when imagining a bistable figure. This is despite the fact that all these participants assigned a different interpretation (i.e., percept) to the figure during direct visual inspection in the memorization phase.

It remains unclear whether providing haptic cues of a bistable figure concurrent with visual imagery of that bistable figure improves successful reinterpretation (as opposed to without haptic support). There was no statistically significant difference in the amount of successful reinterpretation for the visual-haptic condition when compared to the visual only condition, or the haptic control condition. In the next section, we will contextualize the current study, point out some possible shortcomings, and finally conclude with implications.

### Reinterpretation in visual imagery is possible without visual cues

Some previous studies have claimed that reinterpretation in visual imagery is impossible (Chambers and Reisberg 1985; Slezak 1991) whereas others provided evidence in favor of successful reinterpretation. Importantly, the current study replicates previous findings from studies that suggest bistability detection is possible, and distinguishes itself in several ways from these previous studies (Brandimonte and Gerbino 1993; Finke et al. 1989; Hyman and Neisser 1991; Mast and Kosslyn 2002; Peterson et al. 1992).

First, in the current study the bistability example was shown after participants had visually memorized the test figures. Therefore, participants were not led to expect bistability in the test figures during direct visual or haptic inspection in the memorization phase (cf. Brandimonte and Gerbino 1993; Chambers and Reisberg 1985; Finke et al. 1989; Hyman and Neisser 1991; Peterson et al. 1992; Slezak 1991). Second, the current bistability example involved a reversal strategy congruent with the test figures (180° rotation). This congruence ensures a sufficient understanding of the reversal procedure. The current positive finding may, therefore, contrast with studies that deemed reinterpretation in visual imagery impossible, since these studies used exemplars that did not match reversal properties of the test figure (as argued by Peterson et al. 1992). Last, the current study expands on the findings by Mast and Kosslyn (2002), by showing that reinterpretation in visual imagery is possible without visual support. This is an important extension as we can now exclude the possibility that direct visual input during visual imagery is a necessary condition for successful reinterpretation.

### Shortcomings

Several possible shortcomings need to be addressed. First, in a modest amount of trials (11.9%, 19 out of 159 trials) there was premature bistability detection during memorization. It is possible that some participants may have seen bistability during memorization without having reported this (otherwise they would have been excluded). However, this is unlikely if we consider that we base the 30.6% successful reinterpretation rate in the Visual Only condition on participants who did not report (either voluntarily or when explicitly asked after the experiment) seeing bistability of any of the figures during memorization. This strongly suggests that participants were successful in reinterpretation through visual imagery.

Similar to previous research (e.g., Brandimonte and Gerbino 1993; Hyman and Neisser 1991; Mast and Kosslyn 2002), several participants detected both interpretations (e.g., doe and seal are detected during memorization), or the alternate interpretation was dominant (i.e., 26%). This raises potential worries about the robustness of the findings. Is the proportion of participants that can reinterpret an alternate interpretation in visual imagery (30.6% in the visual only condition) based on a reliable estimate? We think this is likely the case because previous studies have obtained strikingly similar rates of reinterpretation (44% in Mast and Kosslyn 2002; 35% for Exp. 1 in Peterson et al. 1992). Furthermore, we have recently gathered additional data (see Pouw, Aslanidou, Kamermans, & Paas, 2017; Exp. 1 in Pouw, Fassi, Aslanidou, Kamermans, & Paas, under review) which in a comparable condition yielded a reinterpretation rate of 20.6% despite the fact that these participants did not receive an example figure of bistability (contrary to the current study which used the old woman/young lady to familiarize participants with bistable figures). Thus, the current study confirms that the estimate of 30.6% detection rate is credible, while excluding possible confounds of premature detection.

Another question that arises out of the current study is why some participants perceive ambiguity during perception while others do not. Unfortunately, the current study cannot directly address this question about the perceptual dynamics of ambiguity detection. However, there is a host of previous research that has shown that both bottom-up and top-down processes are at play in ambiguity detection (for a review see Scocchia, Valsecchi, & Triesch, 2014). For example, it has been shown that size of the figure can affect ambiguity detection (Goolkasian 1991). Once perceived, subjects are able to selectively maintain a percept through focus of attention (Meng and Tong 2004). Moreover, it has been found that creativity measures can predict individual differences in ambiguity detection (Doherty and Mair 2012). To conclude, there is a complex interplay between top-down and bottom-up processes that need to be understood before we can answer why there are individual differences in perception of ambiguity.

It should further be noted that we have used a set of test figures that have not been used in previous research. This yielded unpredicted differences in detectability of bistability as indicated by the statistically significant difference in detection rate for one particular figure. Namely, performance was significantly higher for the swan/elephant in all three conditions (see Table 2). However, this difference in detection rate does not alter the interpretation of the possibility of reinterpretation in visual imagery. Nor is this more readily reinterpretable figure conflating (lack of) differences between conditions, as figures were equally distributed over conditions.

Based on previous research on multimodality, we expected that haptic cues would support the quality of visual imagery, and therefore, increase reinterpretation (Dellantonio and Spagnolo 1990; De Volder et al. 2001; Lacey, Campbell, & Sathian, 2007; Prather and Sathian 2002; Röder and Rösler 1998). The confirmatory statistical analysis suggests that this was not the case when tested within participants who did not detect bistability in any of the three figures during the memorization phase. Given these null-results, we will refrain from interpreting this null-finding. Future studies should ensure sample sizes that are large enough to cope with considerable losses in data that are caused by issues inherent to bistable test figures used in studies on reinterpretation in visual imagery.

We have, however, gathered additional data regarding the possible supportive role of the haptic system in mental imagery (Pouw et al. 2017; Exp. 1 in Pouw et al. under review; preprint [and data] available at https://osf.io/ct4m3/). In this recent study, subjects had to manually explore the visually ambiguous 2-D figures during memorization, without visual perception (similar to our control condition in the present study). First, in the first experiment it was found that a small portion of the subjects that reinterpreted the memorized figures in mental imagery produced gestures (without speech) as if manually exploring the figure. We interpret this finding as an indication that the haptic system may indeed support mental imagery performance. In a follow-up experiment, we obtained that subjects who had to perform a secondary motor task during the reinterpretation of previously memorized figures (drumming their fingers on the table) underperform compared to subjects who do not move, or produce manual movements that follow the contours of the imagined figures. These results are a promising indication pointing toward a functional role of haptic cues in visual imagery processes.

Finally, note that the current study does not provide insight on why participants are (not) able to detect bistability in visual imagery. For example, performance in the present study might be associated with mental rotation abilities (Mast and Kosslyn 2002). Mast and Kosslyn (2002) presented the old woman/young lady as their test figure and found that successful reinterpretation in visual imagery was related to participants’ mental rotation ability. Similarly, our test figures also required an upside down rotation to orient the alternate interpretation. These similarities strongly suggest that mental rotation abilities have played a crucial role in which participants in our sample successfully reinterpreted the figure in imagery. Future research should be especially dedicated in further gauging factors that predict individual differences in reinterpretation performance. However, it may also be possible that differences occur later on in the reinterpretation process. Namely, it is possible that participants were able to retain and successfully rotate the image in visual imagery, but still failed to reinterpret the image because the initial percept (e.g., penguin) is simply too dominant (Chambers and Reisberg 1991). In such a case, participants rotate the original percept and cannot shift their understanding of the image beyond their original percept that now appears upside down (e.g., upside down penguin). As such, further research could focus on individual capacities to ascertain why some and not others are able to reinterpret their visual image, and where such differences occur in the reinterpretation process.

One could wonder if reinterpretation would have occurred in our sample if participants did not receive the instruction to rotate their mental image upside down. In fact, Hyman and Neisser (1991) found that the success of reinterpretation in imagery depends on specificity of the instructions. Their results showed that performance improved significantly depending on how much information was concealed within the instructions—similar to how performance in our sample generally increased with each subsequent question. Therefore, future studies could investigate boundary conditions of ambiguity detection depending on instructions.

## Conclusion

The current results validate previous research and replicates its findings by showing that (a) visual images do not necessarily function as descriptions, and (b) can be used to accomplish similar cognitive acts as with pictorial representations, and (c) bistability detection in visual imagery is difficult (as evidenced by low detection rates). If imagery were to function as descriptions, visual images brought forth from memory do not preserve raw spatial properties of the original source (e.g., duck), rather such spatial properties are encoded under a mode of presentation that is fixed, preventing reinterpretation by an imaginer. Inversely, the current results validate previous research according to which mental images preserve spatial information of an object remembered, and showing that reinterpretations do not need the presence of visual cues.

## Appendix

See Tables 3, 4, 5 and 6.

**Table 3:** Alternatives offered per figure during the multiple choice question

Elephant/swan	Doe/seal	Giraffe/penguin
**(A) Elephant–swan**	(A) Pigeon**–**squid	(A) Crocodile**–**bear
(B) Butterfly**–**eagle	(B) Turtle**–**parrot	(B) Ostrich**–**owl
(C) Jellyfish**–**dinosaur	**(C) Doe–seal**	(C) Monkey**–**otter
(D) Flamingo**–**rhino	(D)Rabbit**–**frog	**(D) Giraffe–penguin**

Alternatives in bold are correct answers

**Table 4:** Correct interpretations based on visually perceived figures during memorization phase

Orientation	Seal/doe	Swan/elephant	Penguin/giraffe
Body orientation	Seal/walrus*	Swan/bird/ostrich/goose/ peacock*	Penguin
Head orientation	Doe/deer/goat/cow/sheep/ giraffe/calf/foal/pig	Elephant/umbrella	Giraffe/bull/cow/deer/ goat*/buck*

Interpretations with asterisk were added post-hoc and based on the judgment of the experimenters

**Table 5:** Performance for natives and non-natives per test condition

Test condition	Native	Non-native
Visual only	8/20 (40%)	3/16 (18.8%)
Visual-haptic	8/19 (42.1%)	10/19 (52.6%)
Haptic control	10/24 (41.7%)	4/19 (21.1%)

**Table 6:** Performance for both orientations of each figure per test condition and question type

Test condition question type	Seal	Doe	Swan	Elephant	Penguin	Giraffe
**Visual only**						
Open question	2/11	0/0	4/5	2/4	1/2	2/14
	(18.2%)	–	(80%)	(50%)	(50%)	(14.3%)
Category hint	2/11	0/0	4/5	0/4	1/2	6/14
	(18.2%)	–	(80%)	(0%)	(50%)	(42.9%)
Multiple choice	5/11	0/0	5/5	0/4	1/2	10/14
	(45.5%)	–	(100%)	(0%)	(50%)	(71.4%)
**Visual-haptic**						
Open question	3/12	2/3	3/6	5/5	0/2	5/10
	(16.7%)	(66.7%)	(50%)	(100%)	(0%)	(50%)
Category hint	3/12	2/3	4/6	1/5	0/2	6/10
	(16.7%)	(66.7%)	(66.7%)	(20%)	(0%)	(60%)
Multiple choice	6/12	2/3	6/6	4/5	2/2	7/10
	(50%)	(66.7%)	(100%)	(80%)	(100%)	(70%)
**Haptic control**						
Open question	1/9	1/5	7/11	1/4	3/10	1/4
	(11.1%)	(20%)	(63.6%)	(25%)	(30%)	(25%)
Category hint	1/9	1/5	8/11	0/4	3/10	1/4
	(11.1%)	(20%)	(72.7%)	(0%)	(30%)	(25%)
Multiple choice	5/9	2/5	11/11	2/4	9/10	1/4
	(55.6%)	(40%)	(100%)	(50%)	(90%)	(25%)

## Footnotes

1 For example, reinterpretation is not directly concerned with whether visual images are experienced as pictures (Thompson 2007). Nor is the question concerned with whether visual images are literally encoded in a visual format (e.g., retinotopic neural representations; e.g., Slotnick et al. 2005), or also co-constituted by bodily re-experiences such as eye-movements (Thomas 1999; Foglia and O’Regan 2015). Nor does the possibility of reinterpretation suggest that top-down propositional processing plays no role in functioning of mental imagery (Langland-Hassan 2015).

2 Note, that the chance that participants perceived bistability during perception was predicted to be relatively low as the figure required a reversal in the vertical plane (i.e., a rotation). For example, Mast & Kosslyn (2002) showed that even when participants are given all the time needed to memorize (and thus perceive) the old woman/young lady figure, the greater majority did not perceive the alternate rotated interpretation. This is to be contrasted with bistable figures, such as the Necker cube, which can alternate perceptually within seconds (e.g., Kornmeier and Bach 2005).

3 Notably, participants who reported an upside down interpretation during memorization had already perceived and memorized the correct orientation of the alternate target interpretation. Therefore, these participants did not have to rotate their mental image in the testing phase to detect the alternate interpretation, despite adjusted instructions instructing them to do so. Since mental rotation ability likely plays a key role in reinterpretation in our experiment, preserving these participants could inflate successful reinterpretation rates (Mast and Kosslyn 2002).

4 Note that we did not find statistically significant relationship between native language (i.e., native, non-native) and reinterpretation (open question) in visual imagery, χ^2^ (1, *N* = 36) = 1.89, *p* = .169). See Appendix C for descriptives.
